# Central Mucoepidermoid Carcinoma in the Mandible Mimicking Dentigerous Cyst: A Report of a Rare Case

**DOI:** 10.7759/cureus.53355

**Published:** 2024-01-31

**Authors:** Anupama Menon, K Subadra, Aravind Warrier S

**Affiliations:** 1 Oral Medicine and Radiology, Sri Ramachandra Institute of Higher Education and Research, Chennai, IND

**Keywords:** mandibular radiolucencies, odontogenic cysts, unilocular radiolucency, mucoepidermoid carcinoma, intraosseous

## Abstract

Mucoepidermoid carcinoma is a rare neoplasm of the salivary gland of which the intraosseous variety is commonly observed with a female predilection and the affected side is more commonly in the mandible. It is usually perceived as an asymptomatic swelling that increases in volume over a few months to a year. They more frequently present as a cortical bulging and are mostly discovered as an accidental finding in a routine radiograph as a well-defined unilocular or multilocular radiolucency resembling an odontogenic cyst. The most widely accepted treatment is radical surgical resection due to its recurrence or metastatic nature. The current case is quite unusual developing in the posterior jaw as a result of an impacted third molar in a 22-year-old female patient.

## Introduction

Salivary gland tumors represent 3-4% of all head and neck neoplasms, with mucoepidermoid carcinoma (MEC) being the most common type followed by adenoid cystic carcinoma [1]. The central or intraosseous variant of MEC is a very rare presentation constituting about 2-3% of MECs, in the fourth to fifth decade of life with a female predilection. Central MEC is an epithelial tumor more likely to originate within the bone or from the epithelium lining of odontogenic cysts or tumors [2]. Radiographically, it is unlike other malignant tumors of the jaw and mimics odontogenic cysts or tumors. The treatment of choice is wide local excision and may require adjuvant radiotherapy in high-grade MEC [3]. This report presents a very rare case of central MEC in the mandible originating from the cystic lining of a dentigerous cyst.

## Case presentation

**Figure 1:**
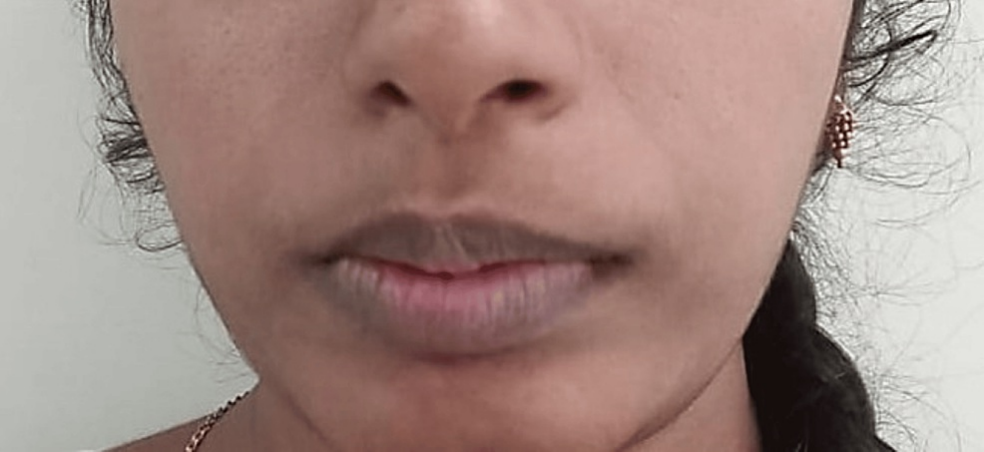
Extraoral examination showing no facial asymmetry

**Figure 2:**
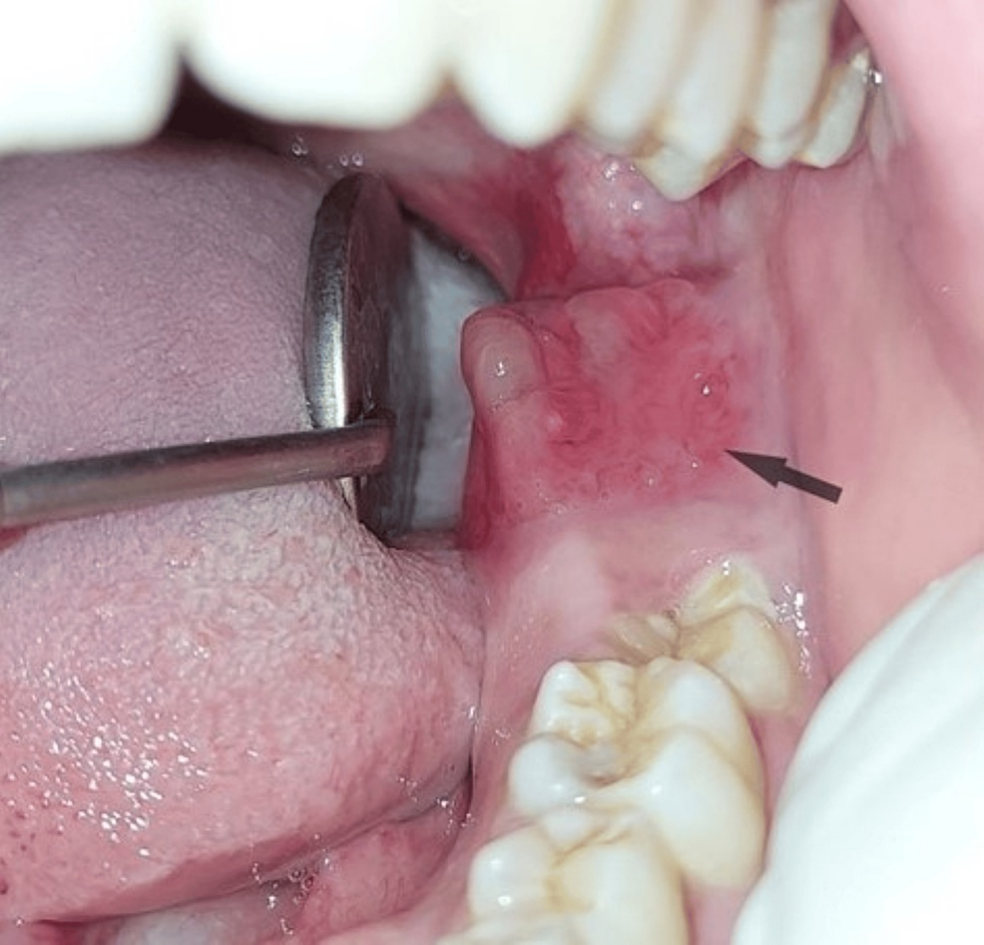
Intraoral bucco-lingual expansion with inflamed alveolar mucosa showing clinically missing left mandibular third molar and partially erupted left mandibular second molar

A 22-year-old female patient was referred to the Department of Oral Medicine and Radiology for an incidental finding of pericoronal radiolucency in the mandibular region of 38 during a routine panoramic examination conducted prior to orthodontic treatment. The patient gave a history of pain in the affected region for four months, followed by a swelling in the alveolar mucosa of the left mandibular third molar region for two months. Her past medical and dental histories were non-contributory. Extraorally, there was no facial asymmetry, and no palpable lymph nodes were noted (Figure 1). Intraorally, there was a bucco-lingual expansion with a partially erupted left mandibular second molar, and clinically missing left mandibular third molar with inflamed and enlarged alveolar mucosa over the same region represented in Figure 2, which was soft in consistency and mildly tender.

**Figure 3:**
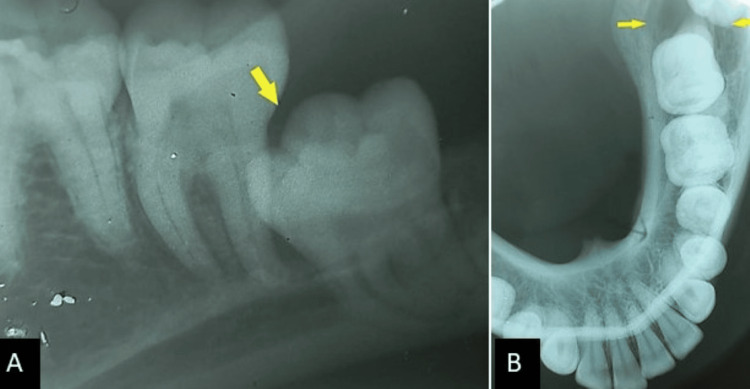
(A) Periapical radiograph reveals pericoronal radiolucency with apical displacement of left mandibular third molar; (B) Occlusal radiograph reveals radiolucency in left mandibular third molar with bucco-lingual expansion.

**Figure 4:**
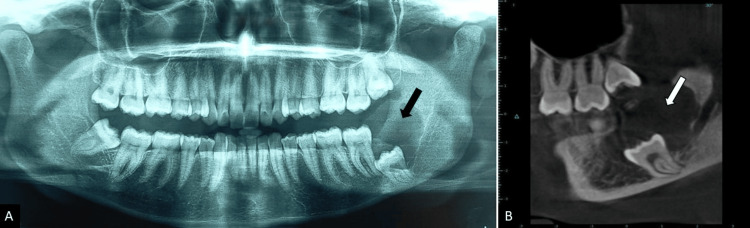
(A) Panaromic radiograph reveals unilocular well-defined pericoronal radiolucency in left mandibular third molar with corticated borders and migration of the mandibular canal inferiorly; (B) Similar features in sagittal section in CBCT imaging CBCT: cone-beam computed tomography

On radiographic examination, the periapical radiograph revealed evidence of a well-defined pericoronal radiolucency with displacement of the left mandibular third molar involving the inferior alveolar canal (Figure 3A). The occlusal radiograph demonstrated bucco-lingual expansion in the left mandibular third molar region (Figure 3B). The panoramic radiograph revealed evidence of a unilocular well-defined pericoronal radiolucency in the left mandibular third molar with corticated borders and migration of the mandibular canal inferiorly (Figure 4A). The cone-beam CT (CBCT) measuring 23x15x19 mm additionally demonstrates relatively comparable findings (Figure 4B). Based on the clinical and radiographic features, a provisional diagnosis of dentigerous cyst with a differential diagnosis of odontogenic keratocyst or unicystic ameloblastoma was made.

**Figure 5:**
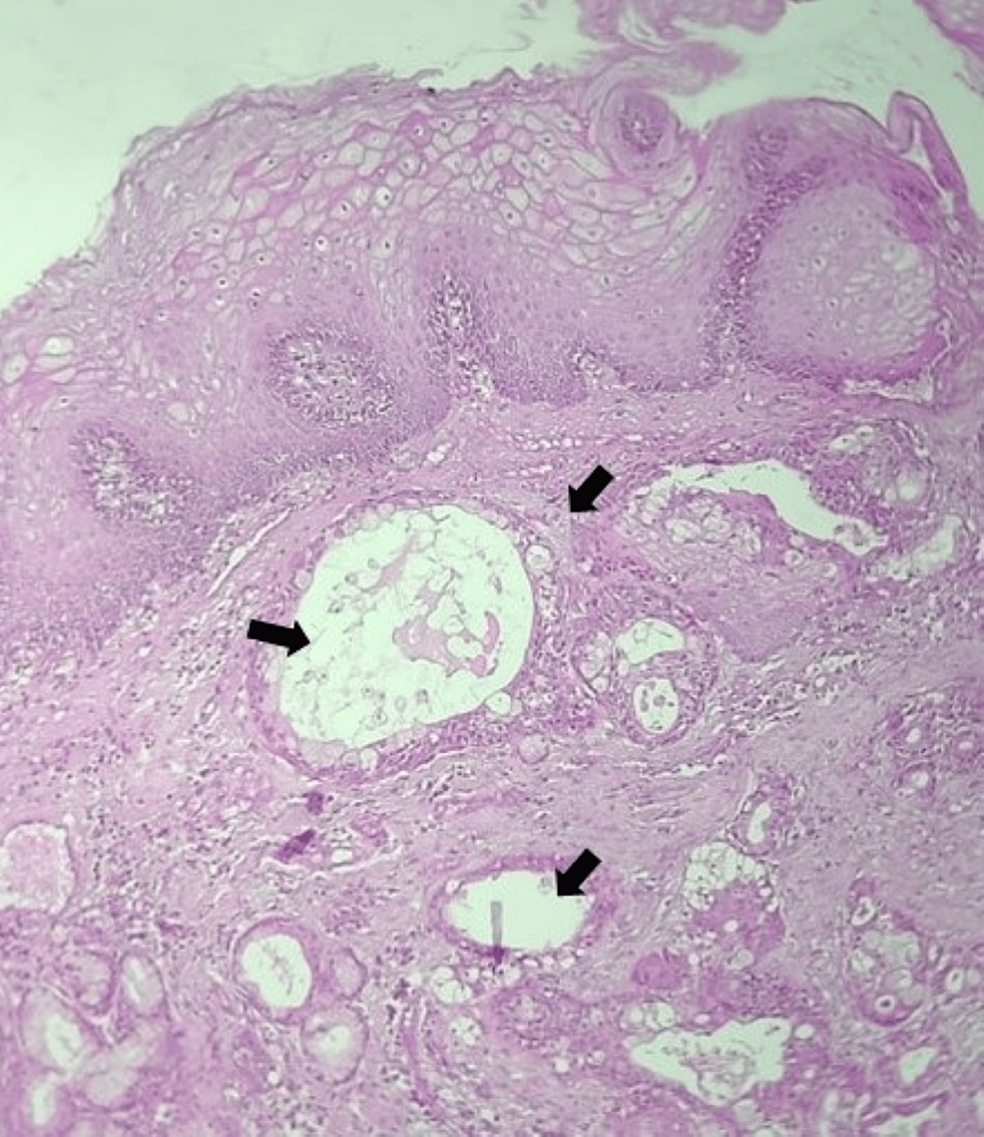
Plenty of cystic spaces, a few of which are filled with mucin, lined by dysplastic mucous cells, intermediate cells, and epidermoid cells

**Figure 6:**
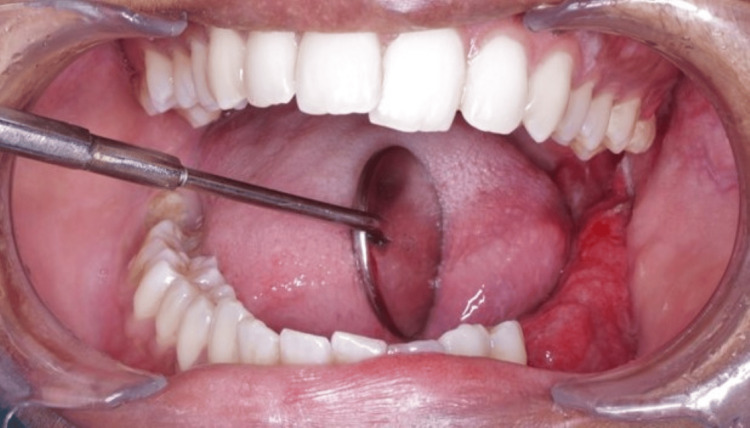
Post-operative intraoral image two weeks after tumor excision.

**Figure 7:**
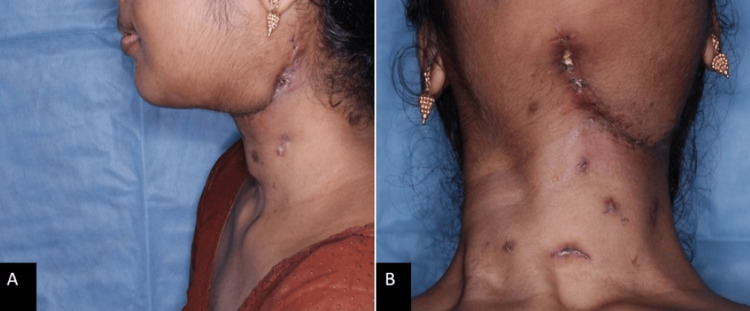
Follow-up extraoral profile of the patient

Further investigation with an incisional biopsy revealed plenty of cystic spaces, a few of which are filled with mucin and lined by dysplastic mucous cells, intermediate cells, and epidermoid cells, suggestive of low-grade mucoepidermoid carcinoma (Figure 5). The patient was subsequently scheduled for a fibula graft-assisted surgical resection of the left mandibular body. Under general anesthesia, a left segmental mandibulectomy with selective neck node dissection and extraction of the left mandibular second premolar was carried out. Following resection, the frozen section was examined histopathologically, and it was discovered to be a low-grade mucoepidermoid carcinoma of the jaw. A fibula transplant was used to reconstruct the deformity following surgery (Figure 6, 7).

## Discussion

Intraosseous MEC is a rare, low-grade neoplasm arising in the posterior region of the mandible. According to literature, it accounts for about 2-3% of MEC cases [2]. The exact etiopathogenesis is unclear, although they have been associated with an origin from an ectopic salivary gland in the form of remnants of salivary glands, metaplastic transformation of odontogenic epithelium, or neoplastic transformation of epithelial lining of the odontogenic cyst [4]. According to Simon et al., intraosseous MEC is more prevalent in females in the fourth to fifth decades of life [5]. In the current case, it is prevalent in the second decade, which is of unique presentation.

Based on the literature, clinical manifestations may include swelling, pain, paraesthesia, and trismus [6]. Our patient who was in her early 20s, reported occasional pain in her left mandibular arch for a period of four months without paraesthesia. The criteria for diagnosis of central mucoepidermoid carcinoma as described by Razavi et al., include the following: (i) intact cortical plates, (ii) radiographic evidence of bone destruction, (iii) histopathologic evidence, (iv) observation of mucin, (v) absence of underlying lesions in salivary glands, and (vi) ruling out metastasis [4].

Based on the literature, the radiographic feature manifests as a unilocular or multilocular, well-defined radiolucency resembling odontogenic cysts or tumors with evidence of root resorption along with dislocation of associated teeth [1,7]. According to Chan et al., these tumors present with a well-sclerotic border and internal sclerotic bone with multiple loculations [2]. In our patient’s case, the radiographic presentation featured a well-defined unilocular pericoronal radiolucency in region 38 with corticated borders and migration of the mandibular canal inferiorly. According to Tapia et al., histopathological examination is required for confirmation of MEC occurring in the form of sheets, ribbons, or islands of mucous cells, epidermoid, and intermediate cells within fibrovascular stroma [7]. In our case, there were epidermoid cells containing dysplastic mucous cells, intermediate cells, and mucin-filled cystic regions. A comparison of the current case with existing literature is given in Table 1 [8-10].

**Table 1:** A comparison of our patient's characteristics with the existing literature

Characteristic Features	Literature	Our Patient
Age group	4^th^ to 5^th^ decade	22 years
Clinical Feature	Swelling, pain, paraesthesia, and trismus	Swelling, pain
Radiographic Feature	Well-defined unilocular or multilocular radiolucency with sclerotic border with or without loculation, root resorption, and displacement of associated teeth	Well-defined unilocular pericoronal radiolucency with corticated border with inferior displacement of mandibular canal
Histopathologic Feature	Sheets, ribbons or islands of mucous cells, epidermoid and intermediate cells within fibrovascular stroma	Cystic spaces filled with mucin, intermediate cells, epidermoid cells with dysplastic mucous cells

For the definitive diagnosis of low-grade intraosseous MEC, a proper correlation of clinical, radiographic, and histological characteristics is essential. When considering the radiographic features, pericoronal radiolucent lesions may resemble odontogenic lesions such as dentigerous cyst, ameloblastoma, odontogenic keratocyst, glandular odontogenic cyst, and adenomatoid odontogenic tumors [8]. A dentigerous cyst is the most common odontogenic cyst seen more commonly in the second to fourth decade presenting as a well-defined unilocular radiolucency with sclerotic border associated with the neck of an unerupted tooth. Ameloblastoma is an odontogenic tumor arising from remnants of the dental lamina, most commonly occurring in the posterior mandible with unilocular or multilocular radiolucency with sclerotic border with displacement of adjacent teeth and root resorption. Odontogenic keratocysts are most commonly seen in the second to third decades in the posterior mandible with a periapical or pericoronal radiolucency with root resorption. Table 2 elaborates on the radiological differentiating features [9-10].

**Table 2:** Distinguishing characteristics arising from several differential diagnoses for the present case.

Characteristic features	Dentigerous cyst	Ameloblastoma	Odontogenic keratocyst	Central MEC
Age group	2^nd^ – 4^th^ decade	Broad age group; commonly seen in 2^nd^ – 5^th^ decade	2^nd^ – 3^rd^ decade	4^th^ – 5^th^ decade
Site	Mandibular and maxillary 3^rd^ molar, impacted canines	Posterior mandible (molar/ angle/ ramus region); may also be seen in maxilla (third molar); anterior (floor of maxillary sinus)	Maxilla and mandible; most commonly seen in posterior mandible (body, ramus);	Mandible (premolar, molar region) more common than maxilla
Radiographic feature	Well-defined unilocular radiolucency with sclerotic border associated with neck of an unerupted tooth (mandibular and maxillary third molars, canines)	Unilocular or multilocular radiolucency; may be separated by septa	Well-defined unilocular radiolucency with corticated border; associated with the crown of an unerupted or impacted tooth	Unilocular or multilocular well-defined radiolucency with evidence of root resorption along with dislocation of associated teeth

The choice of treatment for intraosseous MEC is wide local excision and en-block resection with a margin of adjacent normal bone. Selective neck dissection and radiotherapy are considered in high-grade MECs and to prevent nodal metastasis [2,10]. The prognosis of intraosseous MEC depends on various factors, including sex, histologic grading, surgical treatment plan, and regional nodal metastasis [11]. Mucoepidermoid carcinoma prognosis is determined by the tumor markers Ki-67 and Ck-7, with less than 5% of the index marker indicating fewer recurrences and more than 10% indicating poor clinical outcome [5,12]. Literature indicates that post-surgical recurrence occurs in 40% of cases, with metastasis occurring in 9% of cases [3]. Due to the considerable cost, an immunohistochemistry evaluation was not performed on our patient, who is still being regularly monitored.

## Conclusions

Oral and maxillofacial radiologists have a crucial role in the evaluation and interpretation of radiography images for conditions affecting the face and jaws. This case report is a fine representation of how radiolucencies of the jaw can have a wide range of differential diagnoses, irrespective of whether they have an identifiable radiographic feature. MEC has a better prognosis if it is identified and managed early in its course before challenges emerge.
